# UHMK1-dependent phosphorylation of Cajal body protein coilin alters 5-FU sensitivity in colon cancer cells

**DOI:** 10.1186/s12964-022-00820-8

**Published:** 2022-02-12

**Authors:** Huan Niu, Meng Zhao, Jing Huang, Jing Wang, Yang Si, Shan Cheng, Wei Ding

**Affiliations:** 1 Department of Medical Genetics and Developmental Biology, School of Basic Medical Sciences, Capital Medical University, Beijing, 100069 China; 2 Department of Thoracic Surgery, Beijing Friendship Hospital, Capital Medical University, Beijing, 100050 China

**Keywords:** Cajal body, Coilin, UHMK1, 5-FU resistance, Colon cancer

## Abstract

Resistance to 5-fluorouracil (5-FU) in chemotherapy and recurrence of colorectal tumors is a serious concern that impedes improvements to clinical outcomes. In the present study, we found that conditioned medium (CM) derived from 5-FU-resistant HCT-8/FU cells reduced 5-FU chemosensitivity in HCT-8 colon cancer cells, with corresponding changes to number and morphology of Cajal bodies (CBs) as observable nuclear structures. We found that U2AF homology motif kinase 1 (UHMK1) altered CB disassembly and reassembly and regulated the phosphorylation of coilin, a major component of CBs. This subsequently resulted in a large number of variations in RNA alternative splicing that affected cell survival following 5-FU treatment, induced changes in intracellular phenotype, and transmitted preadaptive signals to adjacent cells in the tumor microenvironment (TME). Our findings suggest that CBs may be useful for indicating drug sensitivity or resistance in tumor cells in response to stress signals. The results also suggest that UHMK1 may be an important factor for maintaining CB structure and morphology by regulating splicing events, especially following cellular exposure to cytotoxic drugs.

**Graphical Abstract:**

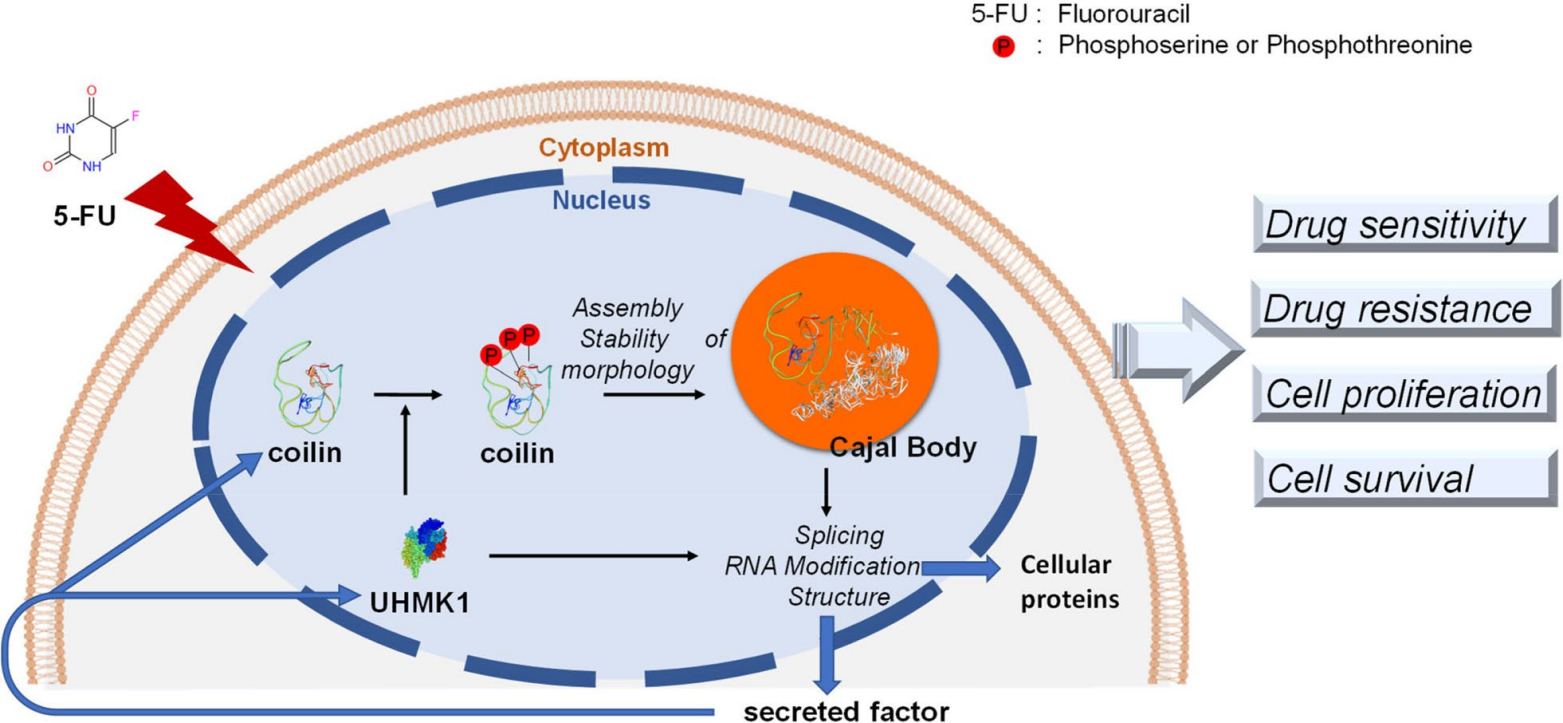

**Video Abstract**

**Supplementary Information:**

The online version contains supplementary material available at 10.1186/s12964-022-00820-8.

## Introduction

Chemotherapy remains the primary option for the treatment of colorectal cancer (CRC), as targeted therapeutic drugs are not well suited for the majority of cases. Increasing evidence from both animal studies and clinical trials implies that drug resistance can develop even during early phases of treatment, resulting in tumor expansion and metastasis [1, 2]. The compound 5-fluorouracil (5-FU) and its derivatives are common chemotherapeutics used to treat a variety of cancers [3], including CRC. Although 5-FU does induce cell death and quickly reduces tumor burden, it is frequently observed in CRC patients that increased levels of apoptosis do not correlate with improved prognosis. In fact, some patients who received adjuvant 5-FU exhibited shorter overall survival compared to those who received only surgical intervention, especially in cases of CRC reoccurrence [4]. Cytotoxic therapy leads to the death of most proliferating cells; nevertheless, cytotoxic pressure has been shown to induce and enrich tumor stem cells with robustness in stemness and plasticity, resulting in acquired drug resistance and the recurrence of cancer [5]. However, the molecular mechanisms that orchestra the development of acquired drug resistance can be very complex. To date, the understanding of 5-FU resistance and strategies to overcome this issue remained to be insufficient to assist clinical applications.

Recent research has focused on the tumor microenvironment (TME) for its vital role in the development of drug resistance, which involves extensive intercellular communication through direct cell-to-cell contact or canonical paracrine pathways [6]. The factors secreted by tumor cells in conditioned medium (CM) include metabolites, cytokines, and growth factors, all of which contribute to changes in cancer cell phenotypes that are similarly observed in vivo [7]. Therefore, in laboratory experiments, culturing cancer cells in CM from defined sources is a commonly accepted approach to discover and dissect the association between TME conditions and inheritable resistance factors.

Cellular responses to CM exposure, especially those related to drug resistance, are often dynamic and chronic processes; hence, morphology indicators are advantageous and useful for technical convenience. Cajal body (CB) is a membraneless organelle observed in different cell types that largely consists of proteins and RNA, and was recently highlighted for its important biological functions [8]. These highly organized nuclear structures in eukaryotes were previously evaluated in studies related to the cell cycle and DNA repair, which revealed the importance and overlap of CBs with the biological processes that define drug sensitivity. In tumor cells, CB morphology has been reported to be a rapid-response index that is extremely sensitive to DNA damages [9]. Coilin is a hallmark protein of CBs and their morphology [10]; number per nucleus, shape, and size were reported as characteristic changes in cell phenotype following DNA damage from treatment with cisplatin [11], daunorubicin [12], and etoposide [13], as well as UV-C or gamma irradiation [14].

As a major scaffold component of CBs, coilin is known to functionally involve many associated nuclear events of RNA processing, including small nuclear ribonucleoproteins (snRNPs) or Cajal body-specific RNPs (scaRNPs). More specifically, biogenesis, maturation, and recycling has been functionally related to coilin, as well as modification of histone pre-mRNAs at the 3’-ends [15]. Previous studies have shown that suppressing coilin decreases cell proliferation and the splicing of certain pre-mRNAs [16]. It has also been suggested that rapid recruitment of coilin to DNA lesions could mediate chromatin conformational changes in response to genotoxic stress. In Coilin^−/−^ mice, defective snRNP biogenesis and splicing exerted a survival problem with reduced viability [17]. Furthermore, stable knockdown of coilin in cells was observed to promote apoptosis and increase chemosensitivity to daunorubicin [18]. However, the molecular mechanism regulating changes in CB formation remains unclear. It has been hypothesized that CBs may directly participate in cellular stress response pathways, participating in certain nuclear events under conditions of drug treatment. Alterations in other essential CB components, such as WD40 encoding RNA Antisense to p53 (WRAP53β) have also been linked to carcinogenesis and associated with poor prognosis of cancer patients [19, 20]. Recent studies have indicated that CB was able to alter the consequence of transcription-related events following DNA damage by changing the dynamics of RNA processing. Aberrant splicing events conferring cancer drug/therapy resistance occur more frequently than previously expected. However, it remains poorly understood how aberrant splicing is influenced in response to environmental stimuli and how this subsequently sculpts cellular phenotypic properties, particularly in highly heterogeneous and progressing malignant tumors.

In the present study, we found that CM derived from drug-resistant HCT-8/FU cells inhibited the sensitivity of native HCT-8 cells to 5-FU, and also induced significant changes in CB morphology. Following a screening with high-throughput transcriptome analysis, we identified UHMK1 as a potential modulator of the adaptative reduction in 5-FU chemosensitivity in response to microenvironment factors in CM. Overexpression of UHMK1 promoted phosphorylation of coilin and altered the formation and reassembly of CBs, resulting in a massive profile change of RNA alternative splicing as well as differential expression of genes responsible for cell growth and survival. Our findings provide an improved understanding of the cancer cell response to environmental stimulation, and further emphasize the importance of Cajal body disassembly/reassembly dynamics for indicating development of an adaptive cancer cell phenotype in a conditioned stress microenvironment.

## Materials and methods

### Tissue culture and cell treatments

The experiments used human colon cancer cells that were 5-FU-sensitive HCT-8 cells and 5-FU-resistant HCT-8/FU cells, which were obtained from MEIXUAN Bioscience & Technology Co. Ltd. (Shanghai, China). The human colon cancer cells SW480 were obtained from the Cell Resource Centre of Chinese Academy of Medical Science (Beijing, China). HCT-8 and SW480 were maintained at 37 °C in a 5% CO_2_ incubator in RPMI 1640 (Invitrogen, Carlsbad, CA) with 10% fetal bovine serum (FBS) (Biological Industries, Kibbutz BeitHaemek, Israel) and 1% penicillin–streptomycin (Keygen Biotech, Nanjing, China). Research grade 5-FU (MCE, Monmouth Junction, USA) was used in the assays to evaluate the cell viability. Apatinib (Hengrui medicine Co, Ltd., Jiangsu, China), raltitrexed (Zhengda Tianqing Pharmaceutical Co., Ltd., Jiangsu, China), artemisinin (National Institutes for Food and Drug Control, Beijing, China), cisplatin (QILU Pharmaceutical Co. Ltd., Shandong, China) were used in the immunofluorescence assays to evaluate the morphology changes of CBs.

### Vector preparation and transfection

The expression plasmid of FLAG tagged UHMK1 were cloned individually into pENTER vectors by Vigene Bioscience Co., Ltd. (Shandong, China). The construct of the UHMK1-K54A mutant was generated from the wild type by Vigene Bioscience Co., Ltd. (Shandong, China). The siRNA targeting UHMK1 duplexes of 5’-AAGCAGUUCUUGCCGCCAGGA-3’ and 5’-CGAGUAUGGUUUCCGCAAATT-3’ were purchased from General Biosystems, Inc. (General Biosystems, Inc, Chuzhou, China). Coilin-targeting siRNA of 5’-GAGAGAACCUGGGAAAUUUTT-3’ was obtained from General Biosystems, Inc. (Chuzhou, China). A scrambled siRNA 5’-UUCUCCGAACGUGUCACGUTT-3’ was used as the control. The cells were transfected with either the UHMK1 plasmids or paired siRNA oligos using a Lipofectamine™ RNAiMAX Kit (Invitrogen, Waltham, Massachusetts, USA) following the vendor’s recommended protocols. Western blotting was performed to detect the protein levels of the corresponded genes at 48 h post transfection.

### Cell viability assay

A number of 5000 cells were seeded in each well of 96-well plates and cultured with 5-FU at 0, 1, 5, 10, 20, 40, 60, 80 μg/ml for 48 h. The cell viability was determined using cell counting kit (CCK8) (KeyGENBioTECH, Jiangsu, China) according to the vendor’s standard protocols. The plates were scanned at 450 nm for absorbance using a spectrophotometer (BioTek, Winooski, VT, USA). Each data point was measured for the average from six duplicates. The experiments were repeated independently for 3 times.

### Immunofluorescence

The method has been widely implemented by our laboratories. Cells of 5.0 × 10^4^ were plated onto a glass coverslip placed into the well of a 12-well plate. The cells on coverslips were fixed, permeabilized, blocked and washed with phosphate-buffered saline (PBS). Anti-coilin (Proteintech Group, Rosemont, USA) was used as the primary antibody and an Alexa Fluor® 594 secondary antibody (Life Technologies, MA, USA) was used for incubation in the dark. The nuclei were stained with Hoechst 33,258 (Sigma-Aldrich, St. Louis, MO, USA) prior to the examination and image acquisition under a confocal system (Leica Microsystems TCS SP8. Wetzlar, Germany). Control samples without adding the primary antibody were prepared for determining the level of non-specific noise.

### mRNA-sequencing and data processing

RNA-seq analyses were performed as earlier described. Cells were scraped off from the surface in trypsin-versene solution and collected by 500 g centrifugation. The pellet was washed with PBS to remove residual media. Total RNA extractions were performed with the RNA-Quick Purification Kit (Yishan Biotechnology Co., Ltd., Shanghai, China) following the manufacturer's protocol. The RNA concentrations were determined using a Nanodrop ND1000 spectrophotometer (Thermo Scientific). The quality assessments and mRNA sequencing libraries were performed in the laboratory of VAHTS Universal V6 RNA-seq Library Prep Kit for Illumina (Vazyme Biotech, Nanjing, China), VAHTS RNA Multiplex Oligos Set1- Set2 for Illumina (Vazyme Biotech, Nanjing, China), VAHTS DNA Clean Beads (Vazyme Biotech, Nanjing, China), VAHTS mRNA Capture Beads (N401-01, Vazyme Biotech, Nanjing, China). All prepared samples subjected to paired-end multiplex sequenced (2 × 150 bp) on the Illumina Hiseq X10 platform. Approximately 8 Gb sequencing data was generated for each sample.

The clean reads in compressed FASTQ format were aligned using HISAT2 (version 2.1.0) to the reference of human genome (Homo_sapiens.GRCh38.dna.primary_assembly.fa) with matched rates over 90%. The resulted SAM files output in the BAM format used with SAMtools (version 1.18, http://samtools.sourceforge.net). The resulted BAM files were sorted with SAMtools. The depth counts were called with HTSeq (version 0.11.2. Linux_x86_64, Simon Anders (sanders@fs.tum.de)) with the reference of human genome (Homo_sapiens.GRCh38.94.gtf), European Molecular Biology Laboratory (EMBL)) used to calculate the Fold change (FC) of FPKM and p value among sample groups according to an over-dispersed Poisson model. Differentially expressed (DE) genes were identified with the thresholds of both 1.5 fold change (|log2FC|≥ 0.58) in mean expression and FDR ≤ 5% using Benjamin-Hochberg procedure. The p-value was identified using DESeq2 (version 1.30.0). The enrichment of DE genes was performed using Gene Ontology (https://go.princeton.edu/) and Cluster 3.0 (Michael Eisen, Stanford University), then displayed with TreeView (version 1.1.6r4, Alok Saldanha). The analyses for alternative splicing events of expressed genes were performed using the rMATS software package (version 4.1.0, http://rnaseq-mats.sourceforge.net/, Xing Lab, Children's Hospital of Philadelphia). The differences in splicing isoforms (SIs) were identified with FDR ≤ 5% and a threshold of *p* ≤ 0.01 in the mean expression between the samples.

### RNA extraction and RT-qPCR

Total RNA was isolated using Trizol (Life Technologies, Carlsbad, CA, USA). HiScript II Q RT Kit (Vazyme, Nanjing, China) was used for reverse transcription. NovoStart® SYBR qPCRSuperMix Plus (Novoprotein, Shanghai, China) was used to quantify gene expression level from the obtained cDNA. The primers for detecting are listed in Additional file 1: Table S1. GAPDH was used as the loading reference. The cDNA was determined using a Quantitative Real-time PCR (Archimed X6, Rocgene, Beijing, China).

### Western blotting

Western blot analyses were performed as earlier described. Briefly, samples of cell lysates were prepared and separated by 10% SDS–polyacrylamide gel electrophoresis (SDS-PAGE), then transferred onto polyvinylidene fluoride (PVDF) filters. The probing antibodies were against the following antigens: UHMK1 (SC-393605, Santa Cruz Biotechnology, Santa Cruz, CA, USA), coilin (10967-1-AP, Proteintech Group, Rosemont, USA) and GAPDH (TA-08, ZSGB-BIO, Beijing, China).

### Co-immunoprecipitation (Co-IP) assay

Cells were harvested and lysed in 1000 µl of ice-cold lysis buffer (10 mM HEPES, 50 mM NaCl, 5 mM EDTA, 1 mM Benzamidine, 0.5% Triton X-100). The lysate was solubilized via end-over-end rotation at 4 °C and clarified via centrifugation at 12,000 rpm for 30 min. A small fraction of the supernatant was taken at this point and incubated with SDS-PAGE sample buffer in order to examine expression of proteins in the whole cell extract. The remaining supernatant was divided equally into two tubes, and then incubated with 2 µg phospho-Ser antibody (SPC-149F, StressMarq Biosciences Inc, Victoria, British Columbia) antibody or 2 µg IgG (C2170, Applygen Technologies Inc, Beijing, China) respectively with end-over-end rotation at 4 °C overnight. After incubated with 30 µl of Protein A/G PLUS-Agarose (sc-2003, Santa Cruz Biotechnology, Santa Cruz, CA, USA) for 3 h with end-over-end rotation at 4 °C, the immunoprecipitated proteins were eluted from the beads with sodium dodecyl sulfate (SDS) sample loading buffer, resolved by SDS-PAGE and subjected to Western blot analyses using an antibody against coilin (10967-1-AP, Proteintech Group, Rosemont, USA).

### Statistical analysis

The analysis of variance (ANOVA) was used to determine the statistical significance of data in multiple groups. The student’s t-test was used to compare cell functions between paired groups. Cases of *p*-value < 0.05 was defined as statistically significant. The program of Prism 8 (GraphPad Software, Inc., La Jolla, CA, USA) was used for data plotting.

## Results

### Cajal body morphology and coilin phosphorylation as indicators of 5-FU sensitivity in colon cancer cells

**Fig. 1:**
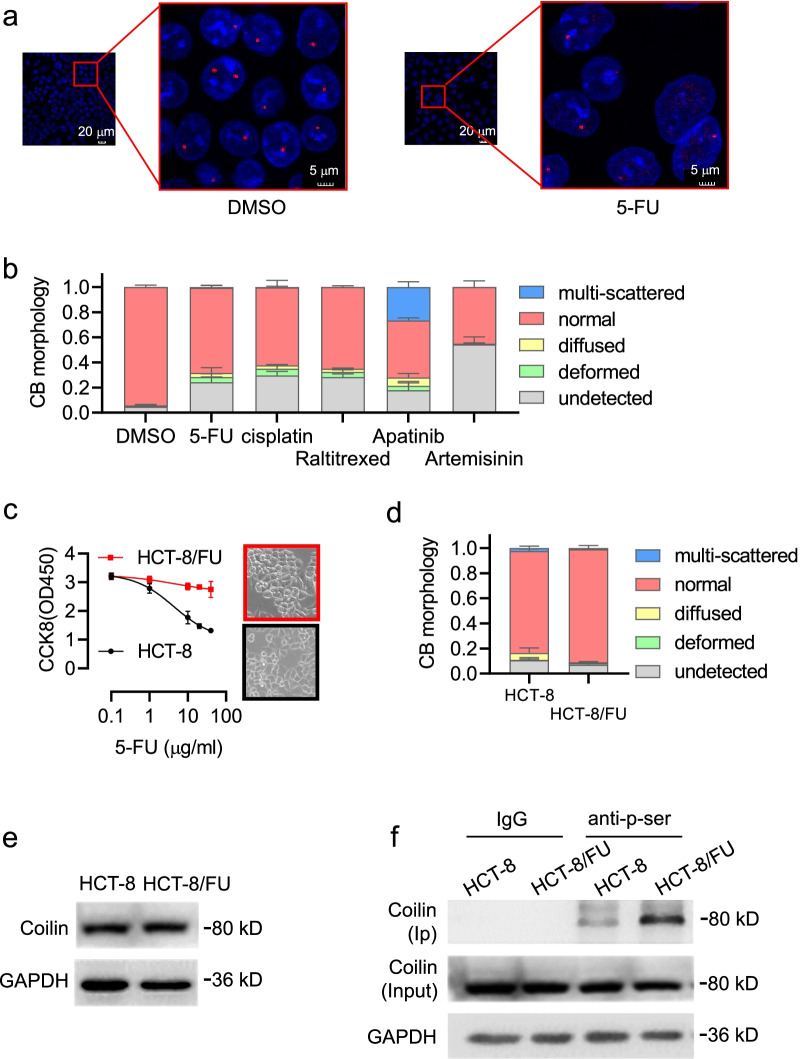
Morphology of Cajal bodies (CB) and coilin phosphorylation in HCT-8 and 5-FU resistant HCT-8/FU cells. **a** Immunofluorescent of coilin in cells treated with 1 μg/ml 5-FU for 24 h. **b** CB morphology in HCT-8 cells following treatments of chemotherapeutic reagents of 5-FU (1 μg/ml), apatinib (20 µM), raltitrexed (2.5 µM), artemisinin (100 µM) and cisplatin (10 μg/ml) for 24 h. **c** Survival of HCT-8 and HCT-8/FU cells treated with 5-FU of increasing doses (0, 1, 10, 20, 40 μg/ml) for 48 h by CCK8 assays. **d** Percentage in counts of various CB morphological categories in HCT-8 and HCT-8/FU cells. **e** Western blots for coilin expression in HCT-8 and HCT-8/FU cells. **f** Determination of coilin phosphorylation in HCT-8 and HCT-8/FU cells

Cajal body morphology, including number, size, and shape, has been reported to change significantly following cell exposure to DNA damaging agents [21]. We observed a significant decrease in CB counts in HCT-8 colon cancer cells following 5-FU treatment, as shown by immunofluorescence of coilin and Survival of motor neuron (SMN), another CB marker protein (Fig. 1a, Additional file 2: Fig. S1a). These changes in CB staining were also observed in HCT-8 cells following exposure to other chemotherapy reagents that are known to cause DNA damage, including 5-FU, apatinib, raltitrexed, penicillin, artemisinin, and cisplatin (Fig. 1b). To determine whether CB morphology can be used as an indicator of cellular chemoresistance, paired 5-FU-sensitive and -resistant HCT-8 cells were analyzed by CCK-8 assay. In the presence of 5-FU, the survived percentage was significantly increased in resistant HCT-8/FU cells compared to the sensitive HCT-8 cells (Fig. 1c). To better describe the changes in CB morphology, we categorized several major types of CB patterns as normal, deformed, diffused, or multi-scattered (Additional file 2: Fig. S1b). Comparing the paired 5-FU-resistant and -sensitive colon cancer cell lines (HCT-8/FU and HCT-8, respectively), we found significant differences in CB morphology in various categories (*p* < 0.01) (Fig. 1d). Coilin acts as a scaffold for the correct assembly of CBs; therefore, both aberrant expression and modification of coilin could influence the formation of CBs [22, 23]. We observed no significant difference in coilin expression at the protein level between HCT-8 and HCT-8/FU cells (Fig. 1e). However, coilin phosphorylation detected by immunoprecipitation assays using a phospho-Ser antibody was significantly increased in HCT-8/FU cells compared to HCT-8 cells (Fig. 1f). These results support previous findings that coilin phosphorylation may be a key factor involved in CB assembly, and may be associated with cell phenotypes related to drug sensitivity and resistance.

### 5-FU sensitivity and CB morphology in HCT-8 cells cultured in HCT-8/FU conditioned medium

**Fig. 2:**
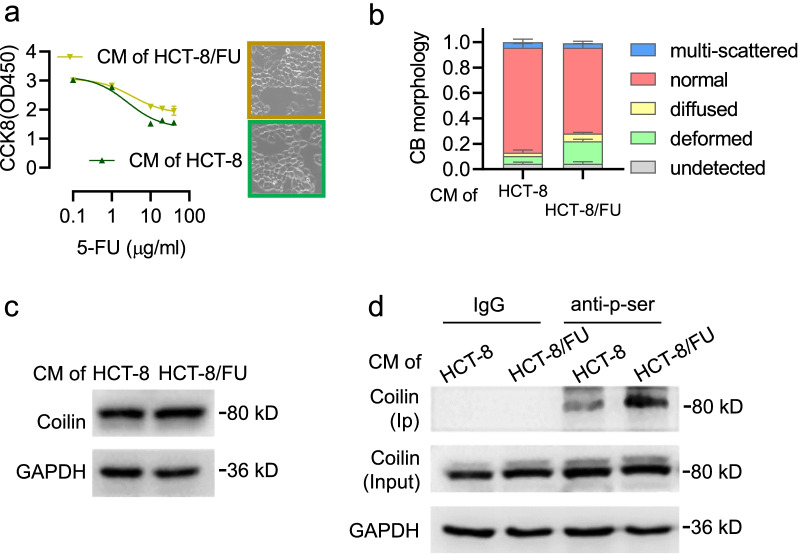
Morphology of Cajal bodies and coilin expression in HCT-8 cells treated with CM from the 5-FU resistant HCT-8/FU cells. **a** Dose responsive results of CCK8 assays in HCT-8 cells treated with CM from HCT-8/FU. **b** Quantitative analyses of CB morphology in different categories. **c** Protein expression of coilin in HCT-8 cells cultured in CM from HCT-8 or HCT-8/FU. **d** Coilin phosphorylation in HCT-8 cells treated with CMs

Recent studies have reported that TME induces a drug-resistant phenotype by transmitting stress signals to adjacent cells and promoting cell survival [24]. Our CCK-8 analysis showed that treatment with HCT-8/FU CM promoted HCT-8 viability in 5-FU dose responses compared to control (Fig. 2a). Aberrant CBs were observed, with changes in both number and shape (Fig. 2b). Coilin protein expression remained stable across groups of HCT-8 cells cultured in CM (Fig. 2c), but coilin phosphorylation was significantly increased in cells treated with HCT-8/FU CM (Fig. 2d). These data indicate that CM derived from drug-resistant HCT-8/FU cells reduced 5-FU sensitivity in HCT-8 cells, highlighting the importance of environmental factors in the development of chemoresistance. In CM-cultured cells, 5-FU sensitivity was once again accompanied by significant CB morphological changes and, interestingly, the changes in counts and percentages of CB types was more significant than that observed in normal cultured cells with similar levels of coilin phosphorylation. This suggests that CB morphology has the potential to serve as a sensitive, rapid, and dynamic indicator of the cellular response to environmental stimuli.

### Transcriptome analyses and validation of UHMK1 involvement in coilin phosphorylation

**Fig. 3:**
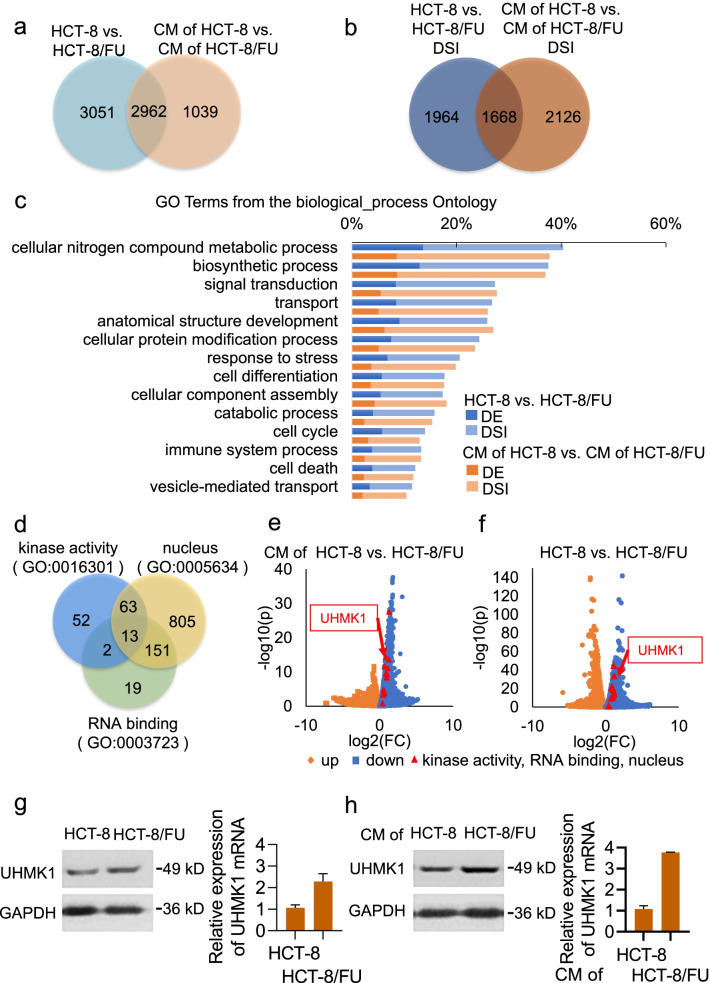
RNA-seq analyses for differentially expressed (DE) genes and alternative splicing events comparing HCT-8/FU cells and HCT-8 cells in both normal culture and CM treatment from HCT-8/FU cultures. **a** Venn diagram illustrating the total and shared numbers of DE genes. **b** Venn diagram summarizing the number of differences in identified splicing isoforms (SIs). **c** Enrichment from Gene Ontology for biological processes on DE genes and differential SIs. **d** Venn diagram on gene sets of kinase activity (GO: 0016301), RNA binding (GO: 0003723) and nucleus (GO: 0005634). **e** Volcano plots and identification of DE genes between HCT-8/FU and HCT-8 cells. **f** Volcano plots of DE genes between HCT-8 treated with CM of HCT-8/FU and CM of HCT-8. **g** Protein (left) and mRNA (right) expression of UHMK1 in HCT-8 and HCT-8/FU cells. **h** UHMK1 expression in HCT-8 cells treated with CM from HCT-8 and HCT-8/FU

The importance of coilin phosphorylation in the formation and stabilization of CB structure has been previously demonstrated [25]. However, the kinases responsible for the phosphorylation of coilin have not been fully characterized, especially in the context of drug pressure. We used an RNA-seq approach to search for possible candidate serine kinases that phosphorylate coilin. Paired 5-FU-sensitive and -resistant HCT-8 cells were compared in both conventional and CM culture conditions. The MA plot was used to show differentially expressed (DE) genes with fold changes (FC) attributable to a given variable over the mean of normalized counts (Additional file 2: Fig. S2a, b). After correction for multiple testing (FDR ≤ 5%), we identified 6,013 DE genes between HCT-8/FU and HCT-8, and 4,001 DE genes between HCT-8 with or without HCT-8/FU CM treatments, using the FC > 1.5 (|log1.5FC|≥ 0.58) as cutoff thresholds (Fig. 3a). A total of 2962 genes were identified in both comparisons; most of these genes were enriched in cell differentiation, response to stress, cell cycle, cell death, and cell proliferation by gene ontology (Fig. 3c). Twelve genes of different abundance were selected and subjected to RT-qPCR analysis, and the results showed a confirmation rate of 68.8% (Additional file 2: Fig. S2c). It was not surprising that a single, known serine/threonine kinase did not stand out as the unique prominent candidate of over-representation.

In addition to DE analysis, we also performed a comprehensive protocol supplied with rMATS software package to identify alternatively spliced transcripts between paired experimental groups [26]. rMATS provides functions to detect, quantify, and visualize complex SIs, including de-novo variations. A skipped exon was the most common event (nearly 70% across the different AS types) followed by intron retention, and mutually exclusive exons, as well as alternative 5' splice site and alternative 3' splice site (each ∼10% across the different AS types) (Additional file 2: Fig. S3a). A total of 3632 differential SIs was identified between HCT-8/FU and HCT-8 cells; a similar number of 3794 was found between HCT-8/FU CM and the control group, including 1668 shared SIs (Fig. 3b). About 30% of the differential SIs were differentially expressed, which is an astonishingly high percentage (Fig. 3c). The differential SIs showed significant enrichment in response to stress, cell differentiation, cell cycle, and cell death; this was in agreement with previous RNA-seq transcriptome analyses, suggesting that the differential SIs may contribute to cell survival (Fig. 3c). We also examined the 12 transcripts for the presence of alternative exons in the coding sequences (Additional file 1: Table S2) and identified eight alternative exons, making up to 76% of the verifiable AS events (Additional file 2: Fig. S3b, c). Coilin has been suggested to have a crucial function in CB-related RNA processing, which is known to affect drug resistance in acute lymphoblastic leukemia (ALL). Therefore, we rationed that reported kinases with activities to induce massive splicing changes may also be a source for identification of potent or novel kinases of coilin phosphorylation, at least in response to 5-FU treatment.

We portraited the properties of a candidate kinase to be: nuclear localization; known substrate of RNA binding or splicing regulator proteins [27]; reported or verified upregulation following drug treatments (especially 5-FU and analogs) [28]; relation to the protein secretion of cancer cells [29]; and/or association with multiple categories as indicated from GO enrichment in the DE analyses of the present study (Fig. 3c), particularly in those that also overlapped with the functional annotation of coilin or CBs. An updated candidate list from the cross-reference of kinase activity (GO: 0016301), RNA binding (GO: 0,003,723), and nucleus (GO:0005634) (Fig. 3d) contained only a limited number of choices (Additional file 1: Table S3). By sifting through our data reports, related database queries, and the literature, the serine/threonine protein kinase U2AF Homology Motif Kinase 1 (UHMK1) was our top selection with the best p value confidence (Fig. 3e, f). UHMK1 mRNA levels were determined by RT-qPCR and confirmed to be upregulated in the paired comparison groups, as shown by RNA-seq (Fig. 3g, h). We also performed Western blotting to detect UHMK1 protein expression, the UHMK1 was found to be upregulated in consistency with findings from the mRNA quantifications (Fig. 3g, h).

### 5-FU sensitivity and coilin phosphorylation in HCT-8 cells following UHMK1 knockdown

**Fig. 4:**
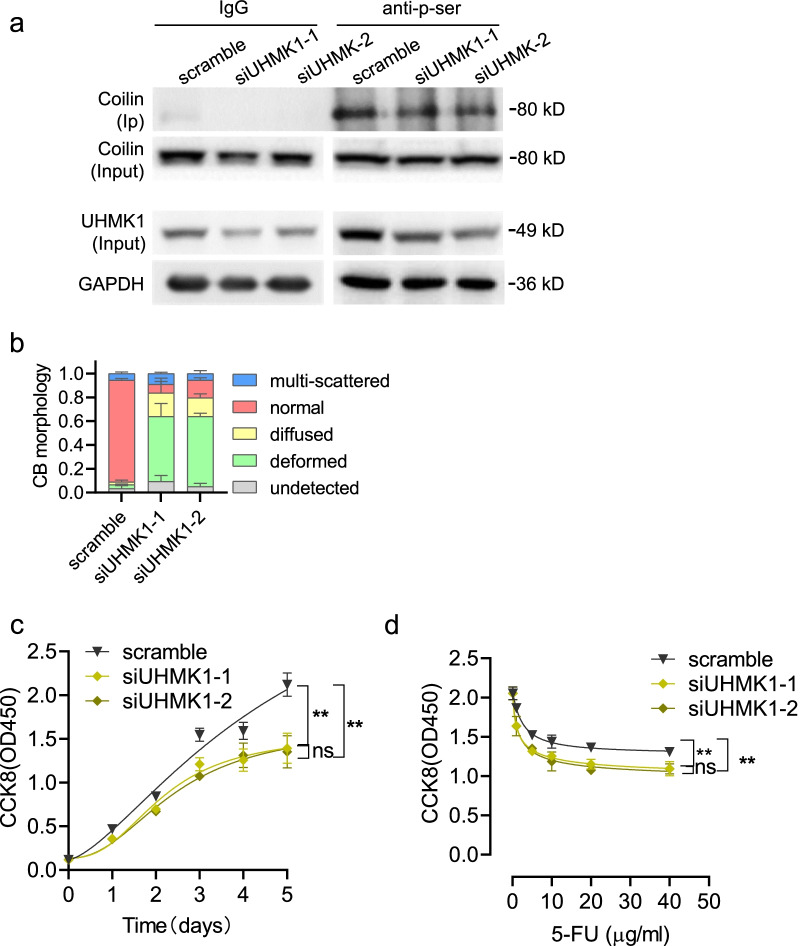
Effects of UHMK1 knockdown on coilin phosphorylation and 5-FU sensitivity in HCT-8 cells. **a** Co-immunoprecipitation assay for coilin serine phosphorylation in UHMK1 knockdown HCT-8 cells. **b** CB morphology and statistics in UHMK1 knockdown HCT-8 cells. **c** CCK8 assays for cell growth of UHMK1 knockdown cells. **d** Survival of UHMK1 knockdown HCT-8 cells following 5-FU treatments (0, 1, 5, 10, 20 and 40 μg/ml) for 48 h. Data were presented as the mean ± SD, n = 6. ** *p* < 0.01

We next aimed to address whether UHMK1 could be functionally involved in the regulation of 5-FU sensitivity in colon cancer cells. Interfering siRNAs against UHMK1 were transfection into HCT-8 cells. UHMK1 expression was decreased compared to scramble controls from the Western blots (Fig. 4a). Immunoprecipitation assays demonstrated that knockdown of UHMK1 significantly reduced coilin phosphorylation (Fig. 4a) and induced significant changes in CB morphology, especially with respect to the percentage of deformed, diffused, and multi-scattered CB types (Fig. 4b). In addition, there cell growth was reduced by ~ 50% in UHMK1 siRNAs transfected cells (Fig. 4c). Cell survival in the presence of 5-FU was also reduced in the UHMK1 knockdown samples compared to scramble controls (Fig. 4d). These data indicate that suppression of UHMK1 expression reduces coilin phosphorylation, alters CB morphology, and influences cell growth and sensitivity to 5-FU in HCT-8 cells.

### Involvement of UHMK1 kinase activity in coilin phosphorylation and associated changes in 5-FU sensitivity

**Fig. 5:**
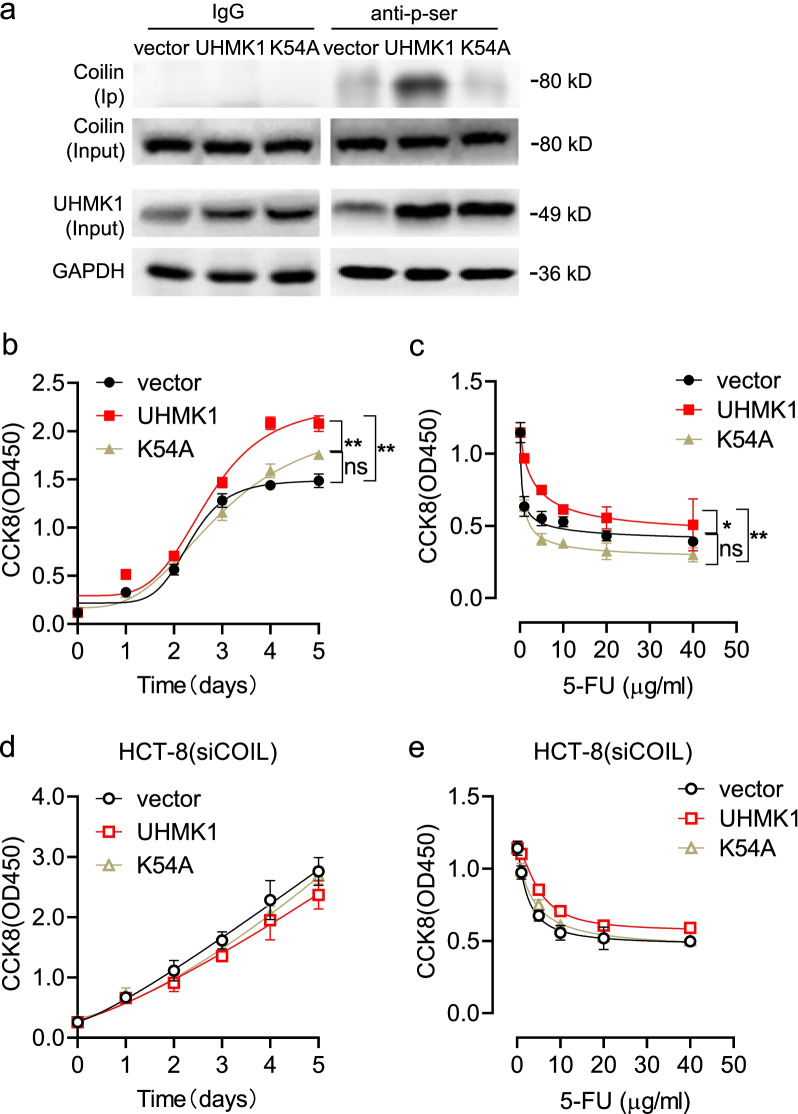
Effects of UHMK1 overexpression in comparison to UHMK1 mutant lacks of kinase activity in HCT-8 cells. **a** Coilin phosphorylation in transfected HCT-8 cells. **b** Cell growth by CCK8 assays. **c** Dose-dependent survival from 5-FU treatments (0, 1, 5, 10, 20 and 40 μg/ml) for 48 h. **d** Cell growth in UHMK1 or mutant transfected HCT-8 cells with coilin knockdown. **e** Cell survival in coilin knockdown HCT-8 cells. Data were presented as the mean ± SD, n = 6. * *p* < 0.05 and ** *p* < 0.01

Since UHMK1 is a serine/threonine protein kinase [30], we deployed a UHMK1 mutant lacking its kinase activity as a dominant-negative agent for transfection into HCT-8 cells to determine whether kinase activity is necessary to mediate the effects on cell responses to 5-FU (Fig. 5a). Increased coilin phosphorylation was observed in wild type UHMK1 overexpression cells compared to vector controls, whereas transfection with the UHMK1-K54A kinase-dead mutant [31] failed to alter phosphorylated coilin levels (Fig. 5a). To further examine whether UHMK1 transfection altered HCT-8 cell growth and sensitivity to 5-FU, CCK-8 assays were performed. Cell growth showed a 160% increase in UHMK1-transfected cells compared to the control (Fig. 5b), and survival of cells was also increased following 5-FU treatment (Fig. 5c). However, no significant changes were observed in HCT-8 cells with UHMK1-K54A overexpression (Fig. 5b, c). To determine whether UHMK1-mediated regulation of cell survival is dependent on coilin expression, we overexpressed UHMK1 in coilin knockdown HCT-8 cells (Additional file 2: Fig. S4). The results showed that the previously observed effects of UHMK1 were abolished in coilin siRNA cotransfection samples (Fig. 5d, e).

### Role of UHMK1 in CB reassembly dynamics during 5-FU drug release experiments

**Fig. 6:**
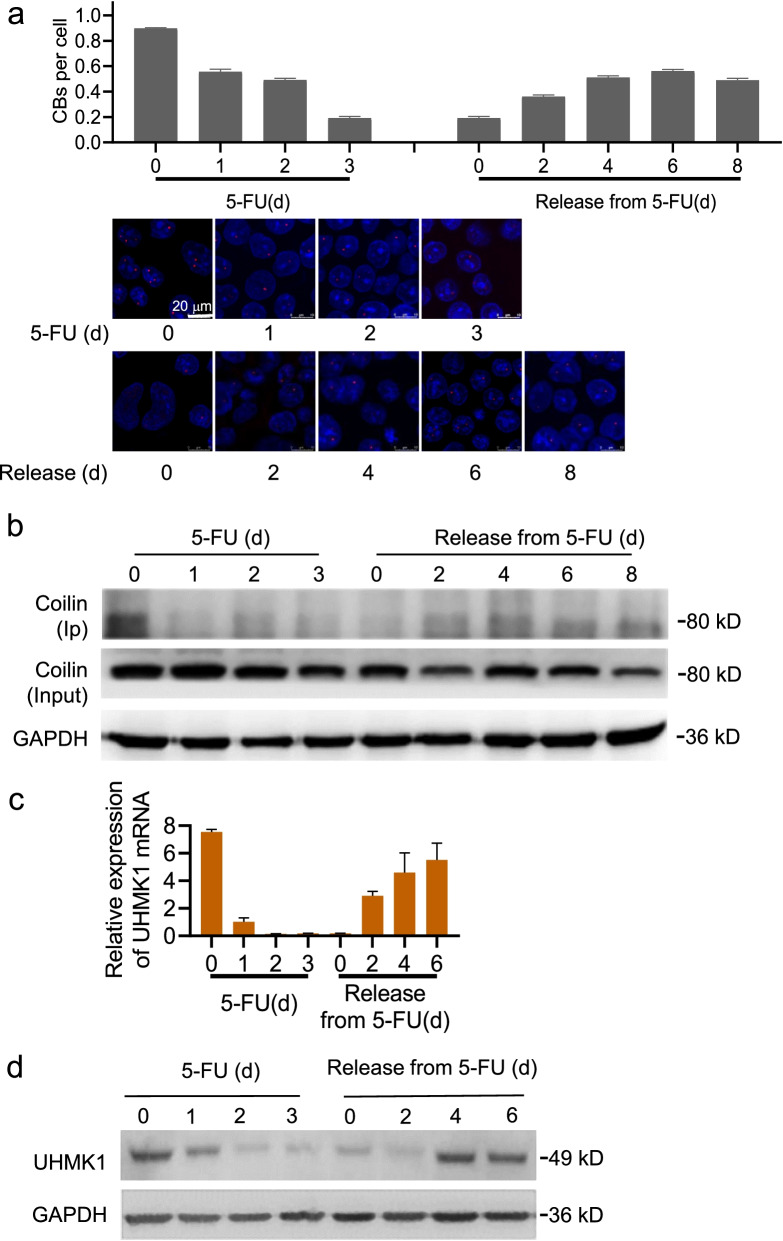
Role of UHMK1 in 5-FU exposure and release in HCT-8 cells. **a** Morphology changes of CBs in 1 μg/ml 5-FU and removal. Significant differences in all the 5-FU drug maintain groups of 1, 2 and 3 days compared to 0 d controls (*p* < 0.01); significant differences in all the release groups of 2, 4, 6 days compared with 0 d group (*p* < 0.01). **b** Western blots for coilin phosphorylation. **c** UHMK1 expression in the HCT-8 cells determined by RT-qPCR. *p* < 0.01 for all the 5-FU drug maintain groups of 1, 2 and 3 days compared to 0 d controls; *p* < 0.01 for all the release groups of 2, 4, 6 days compared with 0 d group. **d** Western blots for UHMK1 expression in the HCT-8 cells

To explore whether UHMK1 has physiological significance in the cellular response to 5-FU treatment and drug-related microenvironment changes, we performed drug release experiments for dynamic observation. The CBs disassembled and decreased in numbers following 5-FU treatment in HCT-8 cells, and this typically progressed during the time course. When 5-FU was removed, CBs reassembled in the surviving cells and eventually recovered to normal counts as well as morphology (Fig. 6a). Throughout the observation period, levels of coilin phosphorylation varied accordingly, while coilin total protein levels remained stable (Fig. 6b). We analyzed protein and mRNA UHMK1 expression in both of the 5-FU drug maintain and release phases across the time course, and found that UHMK1 expression was also correlated with both coilin phosphorylation level and CB count per cell (Fig. 6c, d). These findings strongly suggest that UHMK1 and its serine phosphorylation activity are required factors for CB function in response to 5-FU treatment and are associated with drug-induced cellular stress.

### Coilin phosphorylation, UHMK1, and 5-FU resistance in SW480 cells

**Fig. 7:**
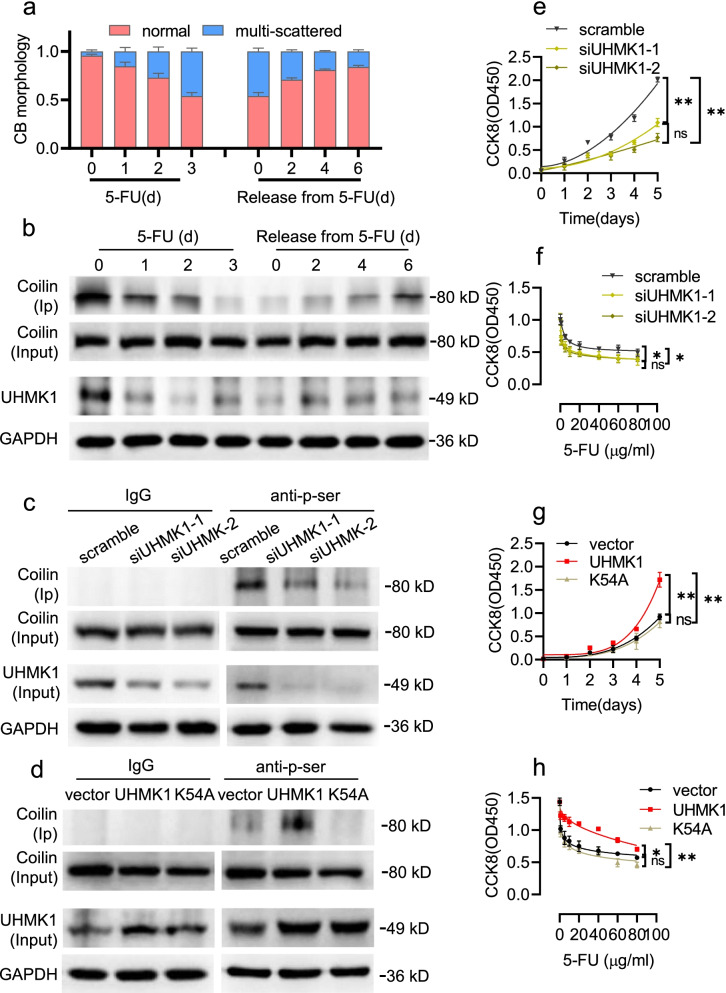
Effects of UHMK1 on coilin phosphorylation and 5-FU sensitivity in SW480 cells. **a** Morphology changes of CBs in 1 μg/ml 5-FU and removal. **b** Western blots for coilin phosphorylation and UHMK1 expression. **c** Co-immunoprecipitation assay for coilin serine phosphorylation in UHMK1 knockdown cells. **d** Coilin phosphorylation in UHMK1 and its mutant transfected SW480 cells. **e** CCK8 assays for cell growth of UHMK1 knockdown cells. **f** Survival of UHMK1 knockdown SW480 cells following 5-FU treatments (0, 1, 5, 10, 20, 40, 60 and 80 μg/ml) for 48 h. **g** Cell growth of transfected SW480 by CCK8 assays. **h** Dose-dependent survival from 5-FU treatments (0, 1, 5, 10, 20, 40, 60 and 80 μg/ml) for 48 h of transfected SW480 cells. Data were presented as the mean ± SD, n = 6. * *p* < 0.05, ** *p* < 0.01

To reinforce our findings in HCT-8 cells, we treated another human colon cancer cell line (SW480) with 5-FU. Following exposure to 5-FU (1 μg/ml) for 3 d, CBs disassembled and decreased in numbers throughout the time course comparing to the 0 d untreated controls (*p* < 0.01). When 5-FU was removed, CBs reassembled in the surviving cells and eventually recovering to normal counts and morphology (Fig. 7a). The expression levels of UHMK1 varied accordingly and correlated well with both coilin phosphorylation levels and CB count per cell across the time course (Fig. 7b). UHMK1 knockdown by siRNAs transfection significantly reduced coilin phosphorylation (Fig. 7c), significantly decreased cell growth, and increased cell sensitivity to 5-FU (Fig. 7e, f). Increased coilin phosphorylation was observed in wild type UHMK1 overexpressed SW480 cells, whereas transfection of UHMK1-K54A reduced the relative levels of coilin phosphorylation (Fig. 7d). Overexpression of UHMK1 significantly promoted cell growth and increased survival of cells following 5-FU treatment (Fig. 7g, h). However, no significant changes were observed in UHMK1-K54A overexpressed SW480 cells (Fig. 7g, h). Moreover, the effects of UHMK1 were abolished in coilin siRNA cotransfection samples (Additional file 2: Fig. S5), which confirmed that UHMK1 regulation of cell survival is dependent upon the level of coilin expression. These data demonstrate that UHMK1 and its serine phosphorylation activity are essential for CB function, and influence cell growth and sensitivity in SW480 cells.

## Discussion

CB size and number can be altered by the cell cycle, development, transformation, temperature, DNA damage, and coilin mutations. Previous studies have suggested that CB morphology appears to be more dynamic in cells with higher metabolic rates, such as neurons and cancer cells. Several recent studies have demonstrated that CB reassembly is sensitive to DNA damage and is an indicator of the repair process in solid tumor cells. In the present study, we found that exposure to CM derived from drug-resistant HCT-8/FU cells reduced 5-FU sensitivity in HCT-8 cells, and also induced morphological changes of CBs (Fig. 2). Secretory factors from resistant cells, including metabolites, cytokines and growth factors in CM, have been shown to confer chemoresistance to sensitive cells through direct cell-to-cell contact or via classical paracrine mechanisms. CBs have the potential to serve as a sensitive indicator for the cell response to cytotoxic pressure, and are also involved in cellular adaptations to environmental stress. Visible coilin protein is known to be phosphorylated at several residues by VRK1 (vaccinia-related kinase 1). VRK1 pathogenic variants have reduced protein stability or kinase activity; functional insufficiency of VRK1 causes a defective formation of 53BP1 (repair) foci in response to DNA damage as well as loss of assembled CBs on coilin in patients with neuromotor developmental syndromes [32, 33]. In the present study, we demonstrated that coilin is phosphorylated at serine residues (Fig. 3) by UHMK1, a serine/threonine protein kinase.

UHMK1 was initially identified to regulate the function of stathmin [34]. Dysregulation or mutations in UHMK1 have been indicated as a high-penetrance factor in human cancers of pancreatic, ovarian, or gastric origin. From public database queries for UHMK1 genetic and epigenetic modifications in colon cancers, we found that gene amplification, nucleotide substitution, and other genomic changes in UHMK1 were associated with poor overall survival in patients (Additional file 2: Fig. S6). In addition, upregulation of UHMK1 to about doubled levels was found in 5-FU-resistant human colon cancer cells in HCT-116 background following increasing dosing of 5-FU for more than 6 months (GSE56322), suggesting the important roles of UHMK1 in the development of chemoresistance. UHMK1 has been shown to bind to a range of proteins, such as eEF1A, FAM64, CDKI, p27KIP1, SF3b155, and CPEB1, demonstrating sophisticated roles for UHMK1 in different cellular contexts [35]. The fact that UHMK1 to regulate a broad range of cellular factors suggests the importance of UHMK1 in RNA processing, nucleotide metabolism, and signaling transduction from secretory granules to the nucleus, which contribute cooperatively to chemoresistance. In this study, we found that UHMK1 enhanced coilin phosphorylation, subsequently regulated CB formation, and contributed to 5-FU resistance in cells (Figs. 4, 5 and 7). Our findings expand our understanding about functional natural substrates of UHMK1 and further demonstrated the importance of coilin phosphorylation in colon cancers, especially in context of drug sensitivity and resistance.

CBs are multicomponent structures that contain a large abundance of splicing snRNPs, suggesting their importance in post-transcriptional RNA modification. The RNA association profile of coilin changes during mitosis compared to interphase, which indicates a function for coilin beyond that of a scaffold protein. Ectopic expression of mutant coilin could induce transcriptional and/or processing dysregulation of a number of CB-related RNA transcripts. Knockdown of coilin was shown to decrease cell proliferation and alter mRNA splicing in minigene reporter assays. These results suggest that the involvement of coilin in splicing regulation may be related to its phosphorylation status, which is also supported by our RNA-seq results that show a large number of differential SIs in 5-FU resistant cells and CM-induced cells (Fig. 3). Aberrant splicing events that confer drug/therapy resistance in cancer are gradually becoming a focus in recent studies, since differential expression of primary transcripts often do not explain the phenotype of drug responses [36]. However, the challenge of profiling SIs and using them for drug sensitivity assessment is not only related to the dynamic and complex nature of splicing events, but is also cumbersome due to a lack of cellular indicators for monitoring the immediate response of cells to drug or environmental stimuli. Fortunately, CBs show potential to serve as a visual maker for evaluating cellular response to cytotoxic pressure and development of resistance to cytotoxic drugs. This notion applies with respect to both environmental stimulation and potential alternative splicing profile changes.

Another interesting finding from this study was that the environmental factors showed a strong ability to change cancer cell resistance to anticancer drugs. Various genetic and epigenetic modifications are the primary ways through which cells cope with environmental stress. Recent studies have demonstrated that cancer cell-secreted molecules are functionally involved in conferring chemoresistance by altering TME. In our study, paired 5-FU-sensitive and -resistant HCT-8 cells were compared to identify factors related to resistance in different genetic backgrounds. CM prepared in vitro from tumor cells of resistant phenotypes has the potential to provide a convenient approach to reconstruct TMEs for laboratory experiments. In such systems, using CB morphology as an index for the evaluation of fast and reversible drug responses may be most advantageous in cultured cancer cells, as it allows for identification of other important kinases (such as UHMK1) responsible for coilin phosphorylation, altering cellular stress response signals, and influencing resistance to chemotherapy drugs.

## Conclusion

In conclusion, we identified that coilin is phosphorylated at serine residues by UHMK1 in colon cancer cells, and this phosphorylation subsequently regulates CB assembly and contributes to colon cancer cell resistance to 5-FU. These findings suggest that coilin may serve as a useful visual maker to indicate cell response under cytotoxic pressure and the development of cell resistance to toxins. CB reassembly is induced by CM from resistant cells or other cytotoxic pressure, and involves complex interactions between snRNAs and snRNP proteins. This leads to spectrum changes in splicing isoforms as well as the selective production of specific SIs that are secreted in TME and remodel the intracellular phenotype of adjacent cells. Our findings suggest a potential mechanism in which TME regulates the development of drug resistance through CB-edited RNA processing, during which UHMK1 is an important participator with crucial functions.

## Supplementary Information

**Additional file 1:** Materials and methods for supplementary data and supplementary Tables (Table S1–S3).

**Additional file 2:** Supplementary Figures (Figure S1–S6).

## Data Availability

Raw data of RNA-seq were available at GEO database repository; accession ID: GSE168888 (all data), GSM5171962 (HCT-8 rep1), GSM5171963 (HCT-8 rep2), GSM5171964 (HCT-8/FU rep1), GSM5171965 (HCT-8/FU rep2), GSM5171966 (CM of HCT-8 rep1), GSM5171967 (CM of HCT-8 rep2), GSM5171968 (CM of HCT-8/FU rep1) and GSM5171969 (CM of HCT-8/FU rep2).

## Abbreviations

5-FU: 5-Fluorouracil
CM: Conditioned medium
CBs: Cajal bodies
TME: Tumor microenvironment
CRC: Colorectal cancers
snRNPs: Small nuclear ribonucleoproteins
scaRNPs: Cajal body-specific RNPs
FBS: Fetal bovine serum
CCK8: Cell counting kit
PBS: Phosphate-buffered saline
Sis: Splicing isoforms
SDS-PAGE: SDS-polyacrylamide gel electrophoresis
PVDF: Polyvinylidene fluoride
CO-IP: Co-immunoprecipitation
SDS: Sodium dodecyl sulfate
ANOVA: Analysis of variance
FC: Fold changes
ALL: Acute lymphoblastic leukemia
UHMK1: U2AF homology motif kinase 1
VRK1: Vaccinia-related kinase 1
