# Supplementary material for: UHMK1-dependent phosphorylation of Cajal body protein coilin alters 5-FU sensitivity in colon cancer cells
Source: Cell Commun Signal. 2022 Feb 12;20:18. doi: 10.1186/s12964-022-00820-8 (PMC8841122; doi:10.1186/s12964-022-00820-8)
Supplement: Supplementary file 2 — Additional file 1: Materials and methods for supplementary data and supplementary Tables (Table S1–S3). [file 12964_2022_820_MOESM2_ESM.docx]

**Materials and methods**

1. Immunofluorescence

The cells on coverslips were fixed with paraformaldehyde and permeabilized using Triton X-100, then blocked and rinsed with phosphate-buffered saline (PBS). The anti-coilin or anti-SMN antibodies (Proteintech Group, Rosemont, USA) were used as the primary probes, and Alexa Fluor® 594 or Alexa Fluor® 488 (Life Technologies, MA, USA) were used as the secondary antibodies for followed incubation in the dark. The nuclei were stained with Hoechst 33258 (Sigma-Aldrich, St. Louis, MO, USA) prior to microscopic examination. The fluorescent images were acquired with a confocal system (Leica Microsystems TCS SP8. Wetzlar, Germany).

### 2. Correlational analysis of gene alteration and expression in colon cancers

To estimate the correlation between UHMK1 gene alteration and expression and colon cancer phenotypes. We analyzed the data from cBioPortal database (TCGA, PanCancer, Atlas) which provides visualization and analyzed results of large-scale cancer genomics data sets. The results are shown as a statistical frequency in categories of gene mutation, amplification and multiple alterations.

**Table S1 The qPCR primers used in this study**

| qPCR primers | Forward 5'-3' | Reverse 5'-3 |
| --- | --- | --- |
| ADAM10 | TGGTGAAACGCATAAGAATCAAT | AACCCAAGCCAGACCAAGTA |
| ANKRD17 | GACATCATGGCAGCAGTCAG | AGGCAAGTGTTAGTGCCGTG |
| ASPM | CACTACAGAGCATTTCTGTCTGC | ATCTCCTCCACATAGCCTGA |
| BCLAF1 | AGCCCTGAAATACACAGGAGA | CGAAGGTCAGCAGAGTCACA |
| CCAR1 | GGCCTTACCACACAAACTCC | CTGAGGTTGTGAATATTGCTGTT |
| CENPF | CCGTCCCCGAGAGCAAGTTTAT | CTGAAACTGCCTTTGCTGCTT |
| CNOT6 | TTAGGCCTGAAAGGAAATCCC | AGACCTTGGAGGTGGTTGTT |
| DEK | TCTCTTCCTTACAGAGAGAGCCAT | CTTCTTTAATGAGGACACAGTGC |
| DLG1 | CCTTGGATCTGGTGTAGGCG | TGCTCTCTGGGTATCTTGCTTC |
| HEATR1 | AAAACTCCGCTGGCGTGAAA | TCCAGGCCAGTACATCCAAT |
| ITGAV | CGCTTCTTCTCTCGGGACTC | AGAAACATCCGGGAAGACGC |
| LRP6 | TCGCCGGTGAGAGAAGAGA | CAAAGGGGCCGCTCTCAGG |
| MKI67 | CGGATCGTCCCAGTGGAAGAG | TCGACCCCGCTCCTTTTGAT |
| PRKAA1 | GGCAAAGTGAAGGTTGGCA | TCTGCGGATTTTTCCTACCACA |
| RORA | ATGGAGTCAGCTCCGGCA | TCGTTACTGAGATACCTCTGCTG |
| TGFBR3 | GGGTCCGGATGGCGTAGTT | CTTGCAATTTTCAAACTGCCTCGGA |
| TOP2A | GTGTCACCATTGCAGCCTGT | ACCAATGTAGGTGTCTGGGC |
| UBR5 | TGGAGGACGAGAAGGAAAGC | TCAGAAACTTCTCGTAACCTGTC |
| XPO1 | AGGAAGGAGCAGTTGGTTCA | CTTGAGCCATTCTTTGCTGGG |
| YAP1 | GAACTCGGCTTCAGGTCCTC | GGTTCATGGCAAAACGAGGG |
| DLG1-3514-1 | TGTTTACAGTTGGTGATATCAGGTGGTGC | CTGCTTCTTCATGAGTAACTTCTTC |
| DLG1-3514-2 | CTAGGTTCCAGTTGGTGATATC | CTGCTTCTTCATGAGTAACTTCTTC |
| YAP1-44800-1 | AGGAAGAGGACCTGAAGCCGAGTTCA | TTCCTGGGGTCCTGCCATGTTGTT |
| YAP1-44800-2 | TCTGGTTCATTGGGGCTGAAGCCGAG | TTCCTGGGGTCCTGCCATGTTGTT |
| DGKA-1651-1 | GATTGGACACCAATGTCCTTCCG | ACACAATGCAGCCCGGTCAG |
| DGKA-1651-2 | TGTGATTGGACGGTTACAGCTCAG | ACACAATGCAGCCCGGTCAG |
| DGKA-16683-1 | TTACAGAGGTTACAGCTCAGTCCC | TGATTGGACCAATGTCCTTCCG |
| DGKA-16683-2 | ACATGTGCTGTCCGTCGTCCTTC | GATTGGACGGTTACAGCTCAG |
| DGKA-16684-1 | TTACAGAGGTTACAGCTCAGTCCC | ATTGGACACCAATGTCCTTCC |
| DGKA-16684-2 | ACATGTGCTGTCCGTCGTCCTTC | GATTGGACGGTTACAGCTCAG |
| DGKA-1665-2 | AGAGGCATCTCCACGCCAATAGA | CAGTTTGTTGAGGTATCACCTCC |
| DGKA-17244-1 | GAGGGTAGGCTGGACTTCTG | CCCTCTCCTTGGCCATCTGTC |
| DGKA-17244-2 | TTCAGAGGGTAGGCCGCTCCAGCT | CCCTCTCCTTGGCCATCTGTC |
| CHTF8-8461-1 | TCCAGCCCTCGCACTGCAAT | CACAGGGATTCCCTCAGTGGTG |
| CHTF8-8461-2 | AGCACAGGGATTCCCTGCAATGGC | CAGTCCTGATCCCCAGGAGTGTGTT |
| ATP5MD-19323-1 | GCCATGATTTCAATCTGGAATGGTGA | TGCATTCACGGAGTTTACAGCTC |
| ATP5MD-19323-2 | CCTGCCATGATTTCAATCTGCCAAAGC | GTTCATTCTACCTGTGAGAGTAT |
| ATP5MD-18693-1 | GCCATGATTTCAATCTGTGTA | GTTCATTCTACCTGTGAGAGTATAA |
| ATP5MD-18693-2 | CATGATTTCAATCTGCCAAAGC | GTTCATTCTACCTGTGAGAGTATAA |

**Table S2 RNA-seq value of selected differential SIs**

|  | Genes | p-Value | IncLevelDifference | Splicing type |
| --- | --- | --- | --- | --- |
| HCT-8/FU vs. HCT-8 | DLG1-3514 | 0.00108 | -0.052 | MXE |
|  | YAP1-44800 | 0.00714 | 0.065 | SE |
|  | DGKA-17244 | 0.00309 | -0.146 | SE |
|  | CHTF8-8461 | 0.00759 | -0.063 | SE |
|  | ATP5MD-19323 | 0.00339 | -0.038 | SE |
| CM of HCT-8/FU vs. CM of HCT-8 | YAP1-44800 | 0.00714 | 0.065 | SE |
|  | DGKA-2566 | 0.00058 | 0.127 | A3SS |
|  | DGKA-1651 | 0.00049 | 0.104 | A5SS |
|  | DGKA-16683 | 0.00061 | 0.186 | SE |
|  | DGKA-16684 | 0.00597 | 0.058 | SE |
|  | ATP5MD-19323 | 0.00339 | -0.038 | SE |
|  | ATP5MD-18693 | 0.00397 | -0.009 | SE |

* **IncLevelDifference = Inclusion Level Difference**

**Table S3 Differentially expressed genes involved in kinase activity (GO:0016301), RNA binding (GO:0003723 ) and nucleus (GO:0005634)**

| **Gene symbol** | **Description** | **HCT-8/FU  *vs.* HCT-8** | | **CM of HCT-8/FU  *vs.* CM of HCT-8** | |
| --- | --- | --- | --- | --- | --- |
|  |  | Log2(FC) | p-value | Log2(FC) | p-value |
| PRKDC | protein kinase, DNA-activated, catalytic subunit [Source:HGNC Symbol;Acc:HGNC:9413] | 1.11E+00 | 1.25E-45 | 6.72E-01 | 2.17E-12 |
| UHMK1 | U2AF homology motif kinase 1 [Source:HGNC Symbol;Acc:HGNC:19683] | 1.15E+00 | 1.79E-34 | 8.50E-01 | 4.66E-15 |
| HSP90AA1 | heat shock protein 90 alpha family class A member 1 [Source:HGNC Symbol;Acc:HGNC:5253] | 8.66E-01 | 5.14E-23 | 5.22E-01 | 2.51E-04 |
| SMG1 | SMG1 nonsense mediated mRNA decay associated PI3K related kinase [Source:HGNC Symbol;Acc:HGNC:30045] | 7.67E-01 | 2.42E-21 | 1.31E+00 | 2.83E-28 |
| PRPF4B | pre-mRNA processing factor 4B [Source:HGNC Symbol;Acc:HGNC:17346] | 1.01E+00 | 1.38E-19 | 9.85E-01 | 4.65E-09 |
| PKN2 | protein kinase N2 [Source:HGNC Symbol;Acc:HGNC:9406] | 1.13E+00 | 2.80E-16 | 1.14E+00 | 8.24E-12 |
| EIF2AK2 | eukaryotic translation initiation factor 2 alpha kinase 2 [Source:HGNC Symbol;Acc:HGNC:9437] | 9.98E-01 | 5.18E-15 | 4.77E-01 | 5.10E-04 |
| CCNT2 | cyclin T2 [Source:HGNC Symbol;Acc:HGNC:1600] | 1.13E+00 | 1.55E-14 | 1.25E+00 | 2.18E-14 |
| ROCK2 | Rho associated coiled-coil containing protein kinase 2 [Source:HGNC Symbol;Acc:HGNC:10252] | 1.02E+00 | 2.05E-11 | 1.03E+00 | 5.32E-10 |
| MAP3K20 | mitogen-activated protein kinase kinase kinase 20 [Source:HGNC Symbol;Acc:HGNC:17797] | 7.91E-01 | 1.36E-10 | 8.59E-01 | 2.45E-10 |
| CPNE3 | copine 3 [Source:HGNC Symbol;Acc:HGNC:2316] | 7.51E-01 | 2.39E-10 | 5.41E-01 | 1.58E-05 |
| FASTKD2 | FAST kinase domains 2 [Source:HGNC Symbol;Acc:HGNC:29160] | 4.28E-01 | 1.35E-02 | 6.55E-01 | 1.18E-04 |
| FASTKD3 | FAST kinase domains 3 [Source:HGNC Symbol;Acc:HGNC:28758] | 4.80E-01 | 1.58E-01 | 4.20E-01 | 1.50E-01 |
