# Supplementary material for: UHMK1-dependent phosphorylation of Cajal body protein coilin alters 5-FU sensitivity in colon cancer cells
Source: Cell Commun Signal. 2022 Feb 12;20:18. doi: 10.1186/s12964-022-00820-8 (PMC8841122; doi:10.1186/s12964-022-00820-8)
Supplement: Supplementary file 3 — Additional file 2: Supplementary Figures (Figure S1–S6). [file 12964_2022_820_MOESM3_ESM.pdf]

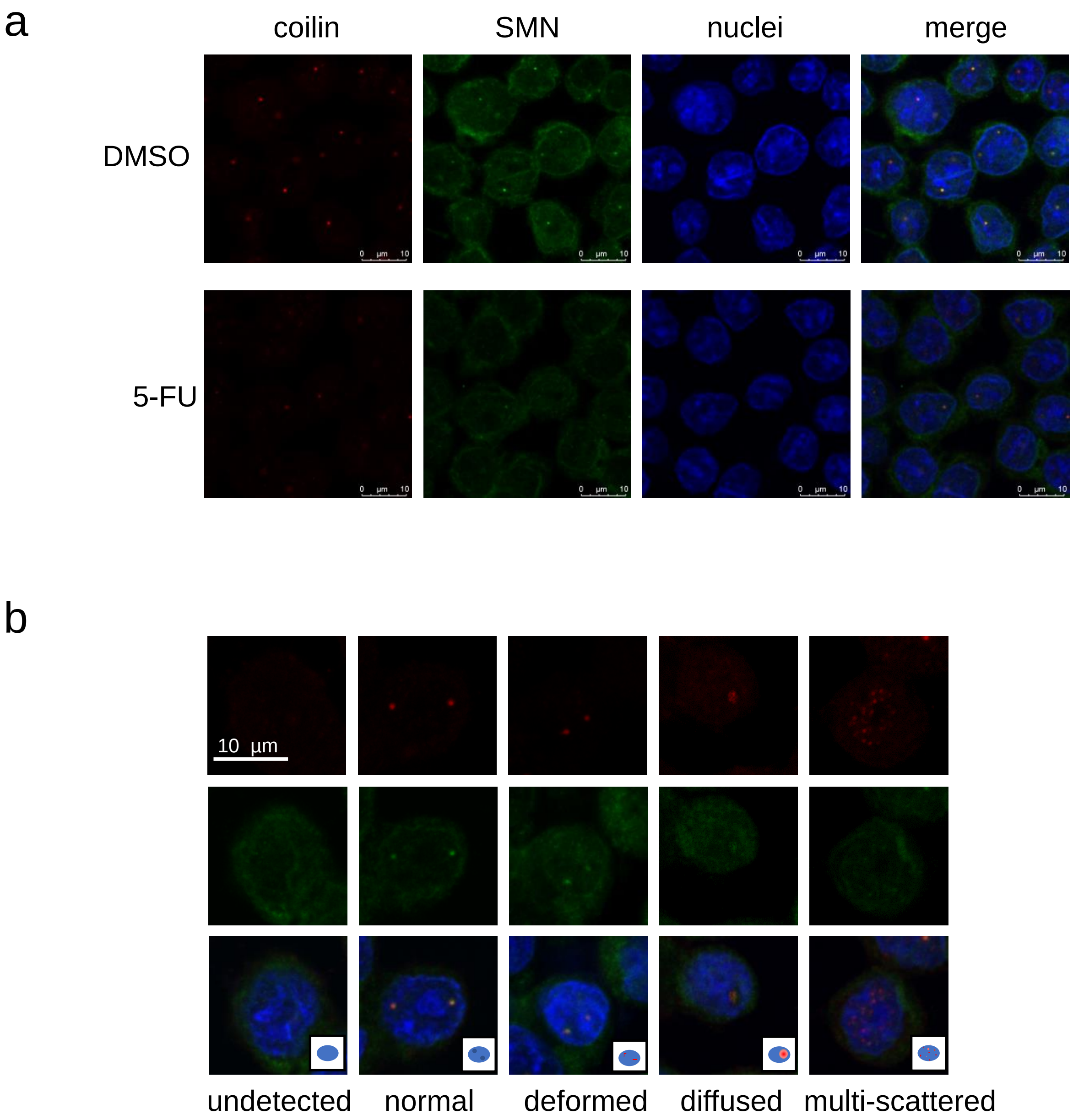

Fig. S1 CB morphologies as from immunofluorescence microscopy. **a** Immunofluorescent staining of coilin and SMN in cells treated with 1 µg/ml 5-FU for 24 h. **b** Types of CBs morphology in HCT-8 following 5-FU treatments. undetected: absent of Coilin staining in the nucleolus; normal: canonical CBs as originally reported; deformed: half-moon shaped or at least 25% variance in diameter measures in CBs; diffused: ambiguous CB structures with above 15% larger area measures; multi-scattered: CBs of disassembled or dispersed morphology in nucleus.

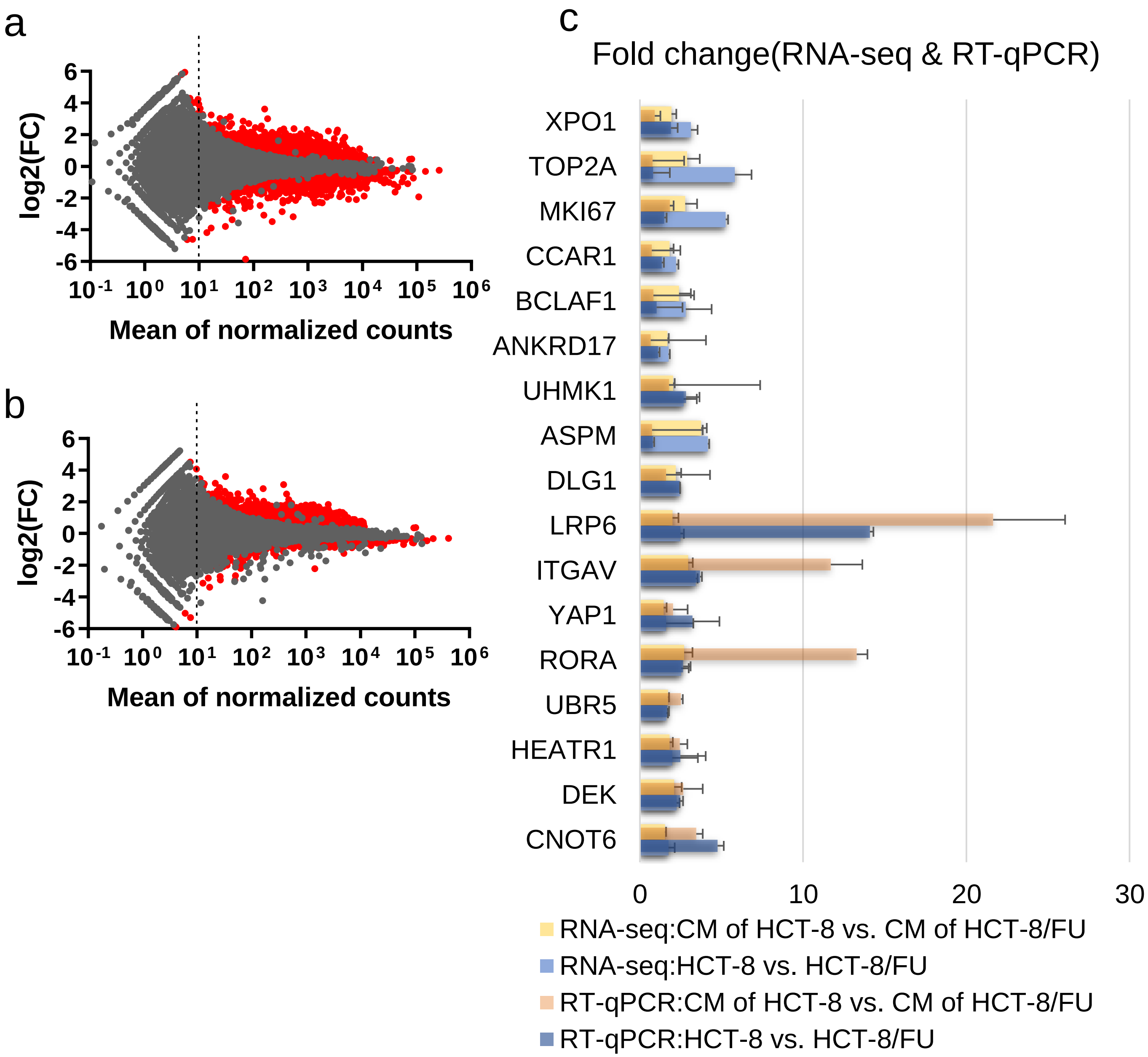

Fig. S2 Transcriptome analysis of HCT-8/FU cells and HCT-8/FU-CM treated HCT-8 cells . **a** MA plot shows the log2-fold change (FC) value attributable to a given gene over the mean of normalized counts for HCT-8/FU cells. Genes with the adjusted p value <0.1 were colored red. **b** MA plot for HCT-8/FU-CM treated HCT-8 cells. **c** Validation of the mRNA levels of selected DE genes by qRT-PCR (mean ± SEM. n=3) and compared with RNA-seq counts.

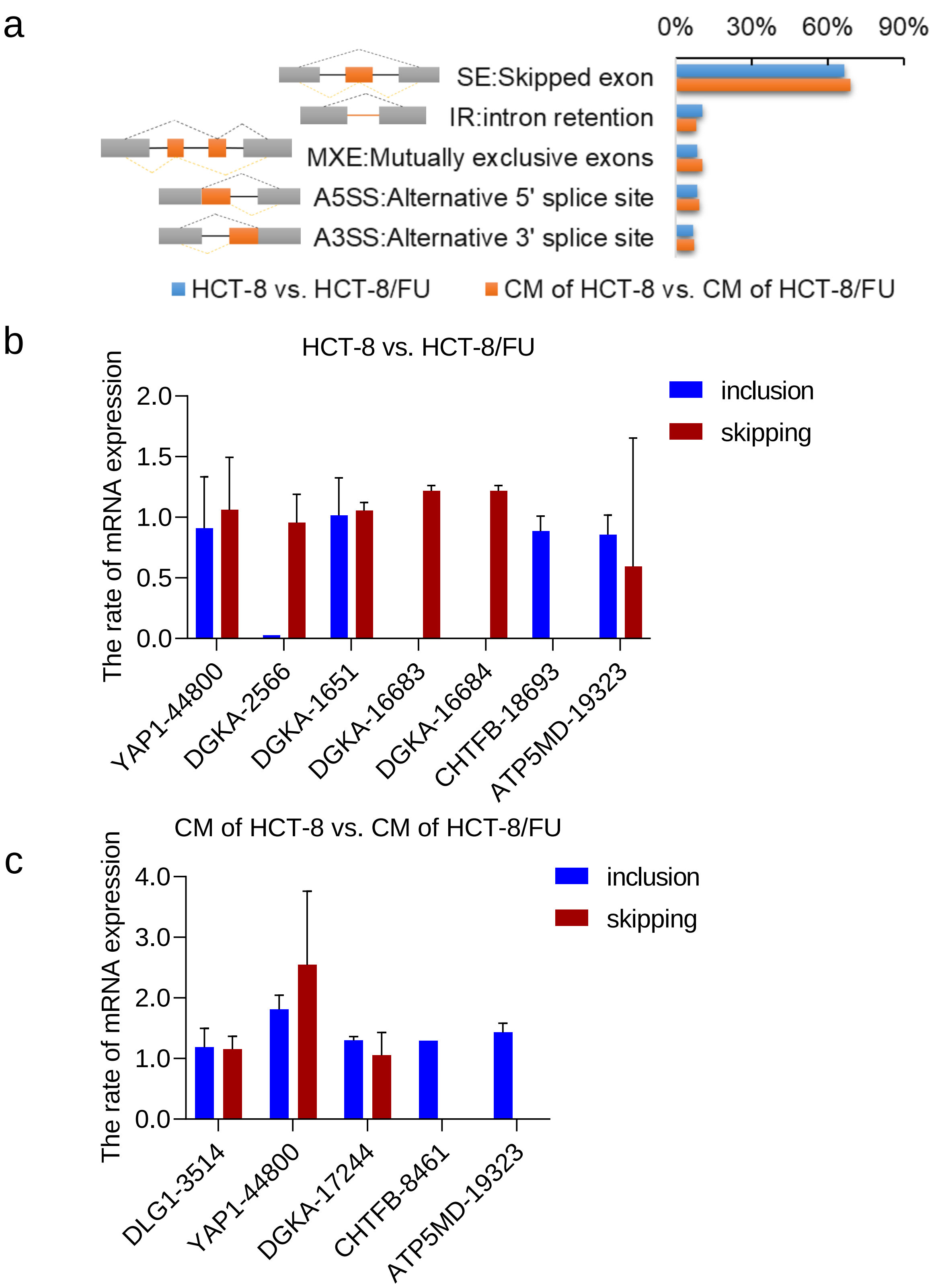

Fig. S3 Alternative splicing analysis of HCT-8/FU cells and HCT-8/FU-CM treated HCT-8 cells. **a** Frequent types of AS events in 5-FU resistant cells and CMs-induced cells. **b** Differential SIs between HCT-8/FU and HCT-8. **c** Differential SIs between HCT-8 treated with CM of HCT-8/FU and CM of HCT-8. Data in e are presented as the mean ± SEM. n=3 per group. *$p < 0.01$.

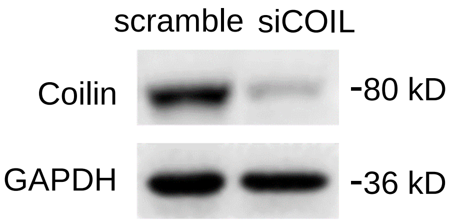

Fig. S4 The expression of coilin in HCT-8 cells transfected with coilin siRNA.

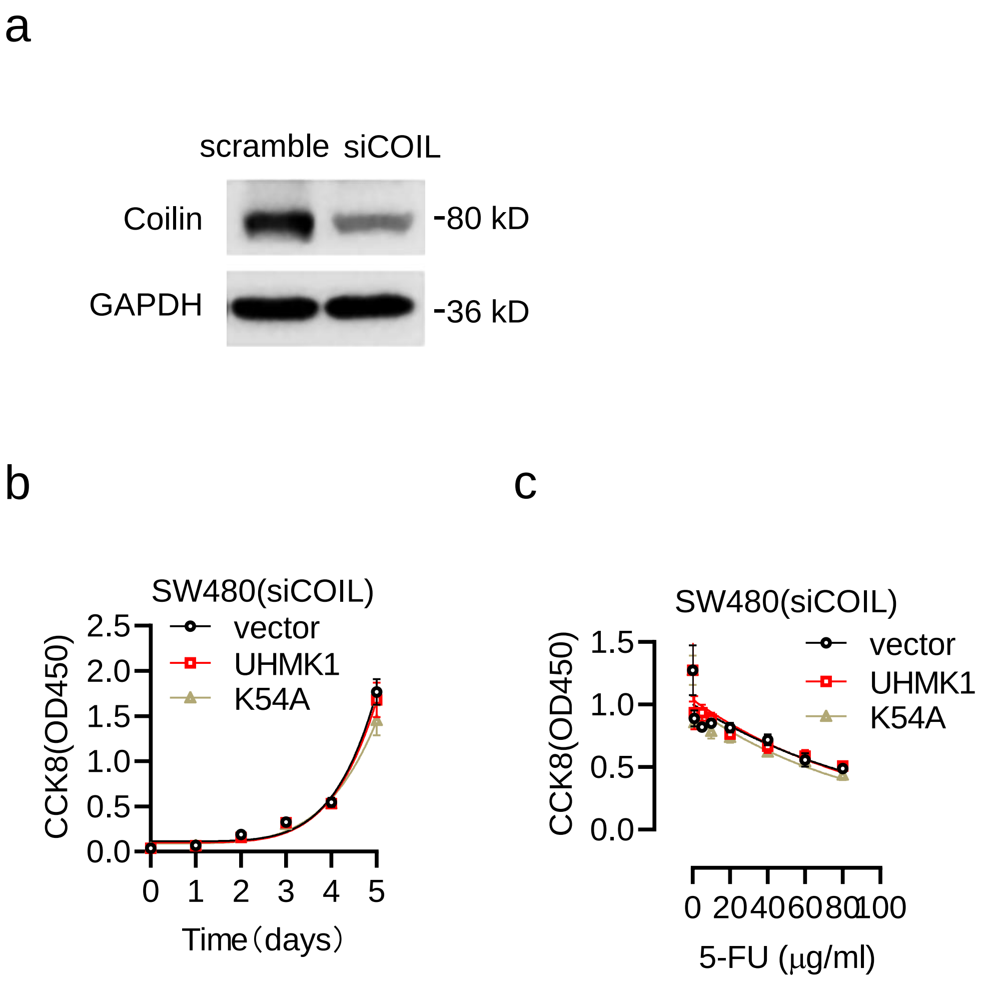

Fig. S5 UHMK1 expression and 5-FU sensitivity were not significantly changed in coilin siRNA transfected SW480 cells. **a** Western blot of coilin at 48 h after transfection of coilin siRNA. **b** Cell growth in coilin knockdown cells transfected with wildtype or mutant UHMK1 overexpression plasmids. **c** Cell survival experiments comparing UHMK1 and K54A mutant transfection. Data were presented as the mean ± SD, n= 6.

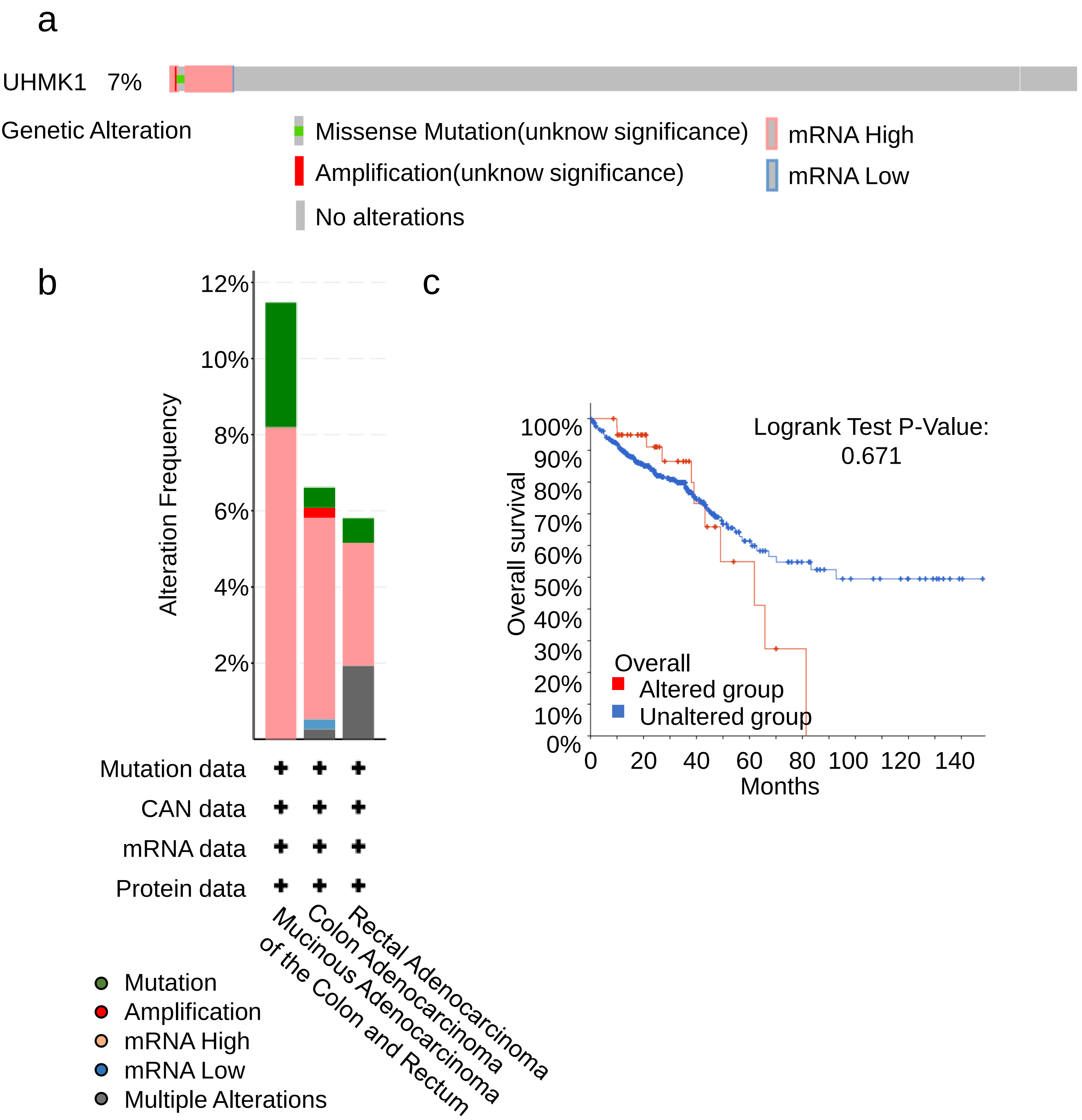

Fig. S6 Genetic alterations detected of human UHMK1 gene in colon adenocarcinoma as documented in the public databases. **a** The data on gene mutation, amplification and multiple alterations of UHMK1 retrieved from cBioPortal as filtered in colon adenocarcinoma (TCGA, PanCancer, Atlas). **b** The statistics of UHMK1 expression in colon adenocarcinoma using data collected in TCGA database. **c** The association of UHMK1 alterations with the overall survival of patients with colon adenocarcinoma.
